# Rectal Surgery

**Published:** 1896-01-18

**Authors:** 


### Progress in Surgery.

### RECTAL SURGERY.

**Treatment of Fistula in Ano by Scraping and Suture.**—Adler[1] states that his experience of this method justifies him in expressing a favourable opinion of it. He thinks its condemnation has been due to a misconception of its range of application rather than to failure to accomplish the desired results in suitable cases. Fistulous tracts running high up into the bowel should not, he considers, be treated by this plan, but only those confined to the lower inch or so of the rectum. In those cases in which primary union was not secured, the immediate suturing of the fistulous tracts prevented hæmorrhage and lessened pain, which is, he believes, occasioned sometimes by the packing of the sinus when sutures are not employed. Adler does not follow the practice of Lange, of New York, and others, in excising the fistulous tract, but contents himself with thoroughly scraping it, and then bringing the sides together with sutures of fishing-gut passed around the sinus, the ends being left long, and the sutures removed in ten days. Wherever the skin or mucous membrane are not closely united superficial sutures are used, in order to prevent the entrance of fæcal matter, which would prove disastrous to primary union. A suppository of iodoform and aristol is inserted into the bowel after the operation. No opium is given, and the bowels are opened on the third day after a copious oil injection into the rectum has been given. The surgeon must be on the outlook for burrowing of pus and the formation of fresh sinuses, and if from the induration of the tissues he concludes that they have formed, he must remove the sutures and open up the sinus with a probe.

**Whitehead's Operation.**—Andrews[2] has collected information from American and European surgeons with regard to the disastrous results which follow Whitehead's operation for piles. He received reports of 200 cases. In eight there was loss of the special sense by which the patient should be warned of a coming evacuation; in twenty-three, incontinence of flatus and fæces; in nine, failure of union of the wound by first intention with retraction of the edges of the mucous membrane forming a contracting tubular ulcer with stricture.

**Ulceration of the Rectum.**—Fraenkel[3] describes nine cases of a particular form of ulceration of the bowel in women, which is not malignant or syphilitic. It usually occurs at a distance greater than 3 or 4 centimetres from the anus. The mucous membrane is eroded over the whole circumference of the bowel, exposing the submucous or even the muscular coat. The base of the ulcer is smooth, and there are no nodules on it. Fraenkel has never seen complete healing, and believes extirpation of the diseased portion of bowel, as performed by Sich and Schede, is the only satisfactory treatment.

**Vaginal Resection of Rectum.**—Rhen advocates resection of carcinomatous disease through the posterior vaginal wall, and has satisfactorily carried out this method in an aged woman with very little difficulty and without much hæmorrhage. An opening in the peritoneum can readily be dealt with.

[1] Medical News, June 1; and Therapeutic Gazette, July 15. [2] Columbus Medical Journal, Vol. XV., No. 3, 1895; and Therapeutic Gazette, Sept 16, 1895. [3] Münchener Medicinische Wochenschrift, June 1895; and Therapeutic Gazette, Sept 16, 1895. [4] New York Med. Record, Oct. 19; and Centralblatt für Chirurgie.

